# Incidence of acute neurosurgery for traumatic brain injury in children—a nationwide analysis from 1998 to 2018

**DOI:** 10.1007/s00701-023-05628-0

**Published:** 2023-05-15

**Authors:** Julius Möttönen, Ville T. Ponkilainen, Grant L. Iverson, Teemu Luoto, Ville M. Mattila, Ilari Kuitunen

**Affiliations:** 1 Institute of Clinical Medicine, University of Eastern Finland, Kuopio, Finland; 2 Faculty of Medicine and Life Sciences, Tampere University, Tampere, Finland; 3 Department of Surgery, Central Finland Hospital Nova, Jyväskylä, Finland; 4 Department of Physical Medicine and Rehabilitation, Harvard Medical School, Boston, MA USA; 5 Department of Physical Medicine and Rehabilitation, Spaulding Rehabilitation Hospital, Charlestown, MA USA; 6 Department of Physical Medicine and Rehabilitation, Schoen Adams Research Institute at Spaulding Rehabilitation, Charlestown, MA USA; 7 Sports Concussion Program, Mass General Hospital for Children, Boston, MA USA; 8 Department of Neurosurgery, Tampere University Hospital, Tampere, Finland; 9 Department of Orthopedics and Traumatology, Tampere University Hospital, Tampere, Finland; 10 Department of Pediatrics, Mikkeli Central Hospital, Mikkeli, Finland

**Keywords:** Traumatic brain injury, Epidemiology, Trauma, Surgery

## Abstract

**Background:**

Most of moderate and severe pTBIs are managed conservatively, but in some cases neurosurgical interventions are needed. The incidence rates of acute pTBI neurosurgery vary considerably between countries and operation types. Our goal was to assess the incidence of acute pTBI neurosurgery in Finland.

**Methods:**

We conducted a retrospective Finnish register-based cohort study from 1998 to 2018. We included all patients that were 0 to 17 years of age at the time of the TBI. The incidence rates of patients with pTBI undergoing neurosurgery and the rates for specific operation types were calculated per 100,000 person-years. We compared the annual incidences with incidence rate ratios (IRR) with 95% confidence intervals (CI). We stratified patients to three age categories: (i) 0 to 3 years of age, (ii) 4 to 12 years of age, and (iii) 13 to 17 years of age.

**Results:**

The total number of neurosurgeries for acute pTBI during the study period was 386, and the cumulative incidence was 1.67 operations per 100,000 person-years. The cumulative incidence during the 21-year follow-up was highest at the age of 16 (IRR 4.78, CI 3.68 to 6.11). Boys had a 2.42-time higher cumulative incidence (IRR 2.35, CI 1.27 to 3.99) than girls (IRR 0.97, CI 0.35 to 2.20). The most common neurosurgery was an evacuation of an intracranial hemorrhage (*n* = 171; 44.3%).

**Conclusion:**

The incidence of neurosurgeries for pTBIs has been stable from 1998 to 2018. The incidence was highest at the age of 16, and boys had higher incidence than girls.

**Supplementary Information:**

The online version contains supplementary material available at 10.1007/s00701-023-05628-0.

## Introduction

Pediatric traumatic brain injury (pTBI) can result in mortality, disability, and further morbidity in children worldwide [3, 11, 37]. Up to 280 per 100,000 children are diagnosed with TBI in emergency departments annually, and pediatric/adolescent age group of 0 to 19 has the second highest incidence of hospital admissions for TBI [11, 26]. In global analysis, higher incidences usually include children 0 to 3 years of age and adolescents 15 to 18 years of age as well as boys after the age of 4 [11]. The most common type of hospitalized pTBI is mild, accounting for at least 81% of pTBI [6, 10, 11]. Studies have reported increasing incidences of mild pTBI in Finland from 1998 to 2018 as well as in the USA from 2002 to 2012 [16, 25, 35]. However, also, decreasing incidences of mild pTBI have been reported [6, 13]. In these countries, the incidence and hospitalization rates of moderate and severe pTBI seem to have remained stable over the years [6, 13, 16, 35].

According to the Brain Trauma Foundations recommendations from 2019, most moderate and severe TBIs are managed conservatively (e.g., ICP-monitoring, hyperosmolar therapy) [23]. In some cases, primary (e.g., evacuation of a subdural or epidural hemorrhage) or secondary (e.g., decompressive craniectomy for intractable intracranial hypertension) brain decompression is required [7, 39, 40]. The incidence of pTBI neurosurgery, regardless of TBI severity, was 4.6 per 100,000 person-years (0.7% of all hospitalized pTBIs) in a nationwide population-based study from Germany [8]. A Norwegian study published in 2021 reported that 16% of moderate and severe pTBIs underwent acute neurosurgery [34]. The most common lesions that require neurosurgical intervention are skull fractures (fracture elevations/repairs 13 to 27% of all neurosurgery), epidural hemorrhages (EDH), subdural hemorrhages (SDH), and subarachnoid hemorrhages (SAH) (evacuations of intracranial hemorrhages 32 to 48% of all neurosurgery) [1, 2, 9, 28, 33, 38]. Insertion of external ventricular drain (EVD) accounted for 34.8% of neurosurgeries in German nationwide analysis [8]. Decompressive craniectomy (DC) has been performed rarely in the pediatric population (0.1% of all hospitalized pTBI from 2014 to 2018) [8]. However, after 2018, recent studies have shown a beneficial effect on the neurological outcome for some selected patients with pTBI [29, 30]. Despite updated guidelines, the role of DC remains controversial. DC has a high risk for complications, and there remains a lack of clearly defined indications for this surgery in children [17, 23].

We have a broad nationwide data with larger sample size and longer follow-up period than in previous studies [1, 2, 9, 28, 33, 38]. We aim to report the trends and the overall incidence of acute neurosurgery for pTBI that has not yet been studied in Finland.

## Methods and materials

We conducted a retrospective register-based nationwide cohort study in Finland. We used two national registers, the Finnish Care Register for Healthcare and the Population Information Register [41, 42]. We gathered the data for the study period from January 1998 to December 2018.

We included all patients with pTBIs, 0 to 17 years of age, from the Finnish Care Register. The register is maintained by the Finnish Institute of Health and Welfare. It contains the patient information for all secondary- and tertiary-level specialized healthcare visits, operations, and hospitalizations in Finland. The register has been shown to be accurate and has thorough coverage [32]. However, the register contains limited information on the severity and injury mechanism of a TBI. We used the International Classification of Diseases 10th Edition (ICD-10) diagnostic codes (S06*) to cover all TBI-related hospital admissions. The rate of TBI-related neurosurgery was based on the Nordic Medico-Statistical Committee Classification of Surgical Procedures codes (Finnish version). The length of the treatment period was calculated from the admission date and reported discharge day. The data regarding the length of hospital stay is somewhat unreliable, because in the case of hospital transfers (e.g., from central hospital to the university hospital), the register treats these as separate treatment periods (the time starts at 0 at the receiving hospital). We calculated the total number of neurosurgeries using the surgical procedure codes and separated patients that had multiple operations during one hospitalization using their unique identification codes and primary injury dates to evaluate the number of cases. The codes did not contain ICP-probe installations because of the unreliability of the number of ICP-probe installation recordings in the Finnish Care Register. We did not include isolated fracture operations because we were unable to verify whether the patients with skull fractures had a concomitant TBI. The full list of diagnosis and neurosurgical procedure codes used in this study is provided in a table in the Appendix (see Appendix 1).

## Statistical analysis

The incidence of neurosurgeries for pTBI was calculated per 100,000 person-years. We compared the incidences with incidence rate ratios (IRR) with 95% confidence intervals (CI) that were calculated using Clopper–Pearson exact method. To evaluate the incidences for different ages, we stratified the ages in 3 categories: 0 to 3 years, 4 to 12 years, and 13 to 17years. We determined the incidence stratified by the operation type: evacuation of intracranial hemorrhage (including evacuation of epidural hemorrhages, evacuation of acute subdural hemorrhages, and evacuation of intracerebral hemorrhages), insertion of an EVD, and decompressive craniectomy. The number of children at the end of the year was used as the denominator in the incidence analysis. We gathered the population information from the open-access register of the Statistics Finland [42]. All statistical analyses were done using R version 4.0.5 [43].

## Ethics

According to the Finnish research legislation, evaluation of the ethical committee is not mandatory for register-based retrospective studies. The study data is pseudonymized with pseudonymization key by Statistics Finland, and none of the authors have access to it. The data was handled in a safe remote-controlled environment that required two-phase identification in every login. The Finnish data authority Findata gave permission to access the Care Register (permission number: THL/2058/14.02.00/2020). Statistics Finland gave permission to access the Population Information and Register of Death Causes (permission number: TK/380/07.03.00/2020). None of our data can be made available according to the law of secondary use of patient information. It is not possible for researchers living outside of Finland to access the Finnish register data.

## Results

**Fig. 1:**
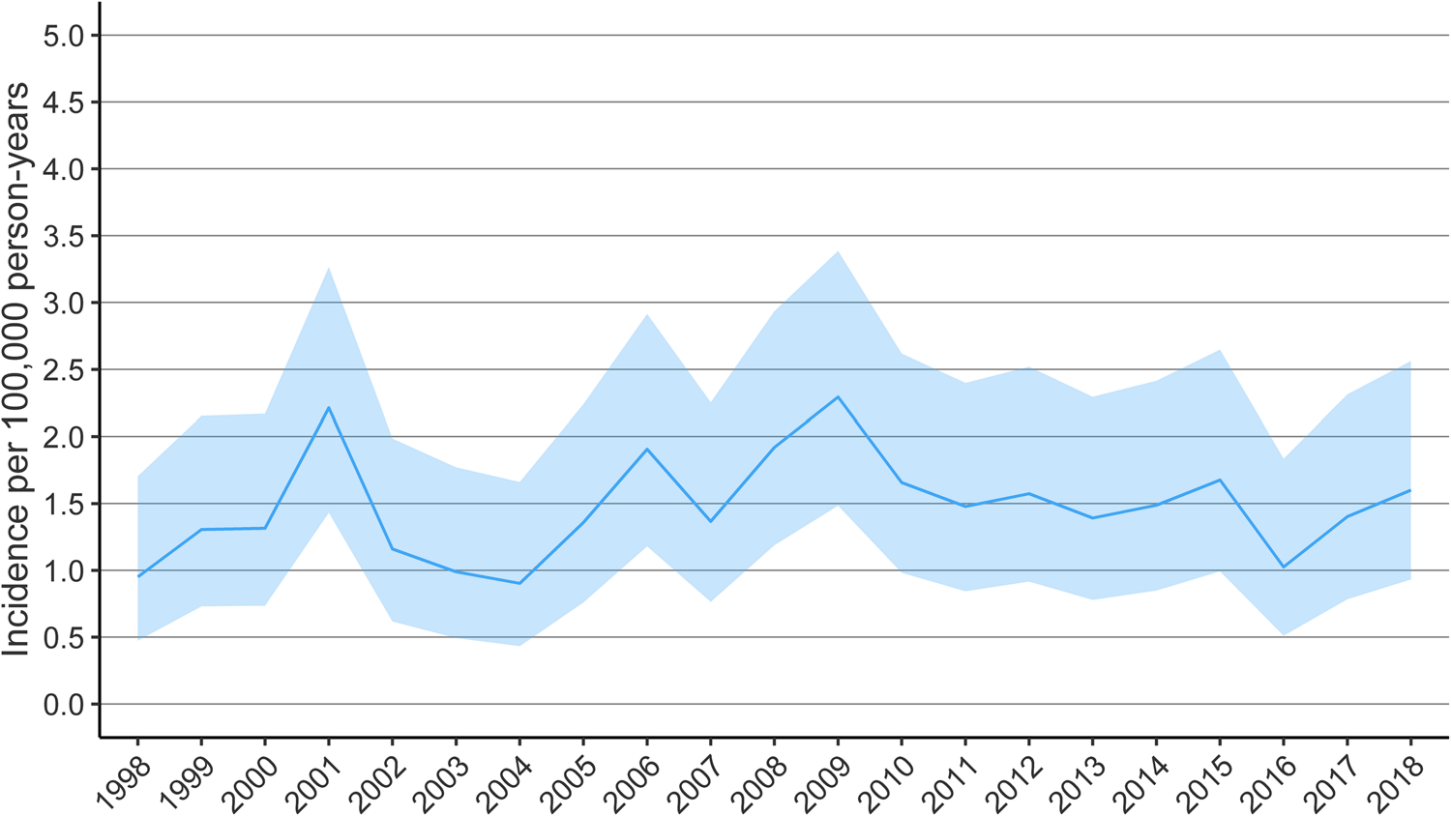
Incidence of neurosurgeries for pTBIs during the years 1998–2018

The incidence of acute neurosurgery for the treatment of pTBI remained relatively stable during the 21-year follow-up. Total number of patients undergoing neurosurgery for pTBI during the entire study period (1998–2018) was 341, and the cumulative incidence was 1.47 per 100,000 person-years. Total number of operations was 378, and 32 patients had multiple surgical interventions. The mean treatment period was 55 days, and median was 24 days. On average, patients underwent 1.11 neurosurgeries, and the median was 1. (see Fig. 1 and Table 1).

**Table 1:** Number and incidence of different operations in different age groups and genders per 100,000 person-years during the 20-year study period

	Boys					Girls				
	*n*	Person-years	Incidence	95% CI lower	95% CI upper	*n*	Person-years	Incidence	95% CI lower	95% CI upper
Decompressive craniectomies										
0–3 years	1	2,502,041	0.04	0.00	0.22	0	2,393,505	-	-	-
4–12 years	9	5,896,214	0.15	0.07	0.29	1	5,650,833	0.01	0.00	0.01
13–17 years	31	3,392,053	0.91	0.62	1.30	14	3,248,508	0.43	0.24	0.72
Total	41	11,790,307	0.35	0.25	0.47	15	11,292,846	0.13	0.07	0.22
Installation of EVD										
0–3 years	9	2,502,041	0.36	0.16	0.68	2	2,393,505	0.08	0.01	0.30
4–12 years	25	5,896,214	0.42	0.27	0.63	7	5,650,833	0.12	0.05	0.26
13–17 years	43	3,392,053	1.27	0.91	1.71	10	3,248,508	0.31	0.15	0.57
Total	77	11,790,307	0.65	0.52	0.82	19	11,292,846	0.17	0.10	0.26
Evacuation of epidural hemorrhage										
0–3 years	23	2,502,041	0.92	0.58	1.38	10	2,393,505	0.42	0.20	0.77
4–12 years	35	5,896,214	0.59	0.41	0.83	27	5,650,833	0.48	0.31	0.70
13–17 years	55	3,392,053	1.62	1.22	2.11	18	3,248,508	0.55	0.33	0.88
Total	113	11,790,307	0.96	0.79	1.15	55	11,292,846	0.49	0.37	0.63
Evacuation of acute subdural hemorrhage										
0–3 years	4	2,502,041	0.16	0.04	0.41	3	2,393,505	0.13	0.03	0.37
4–12 years	6	5,896,214	0.10	0.04	0.22	4	5,650,833	0.07	0.02	0.18
13–17 years	14	3,392,053	0.41	0.23	0.69	6	3,248,508	0.18	0.07	0.40
Total	24	11,790,307	0.20	0.13	0.30	13	11,292,846	0.12	0.06	0.20
Evacuation of intracerebral hemorrhage										
0–3 years	3	2,502,041	0.12	0.02	0.35	3	2,393,505	0.13	0.03	0.37
4–12 years	5	5,896,214	0.08	0.03	0.20	1	5,650,833	0.02	0.00	0.10
13–17 years	7	3,392,053	0.21	0.08	0.43	1	3,248,508	0.03	0.00	0.17
Total	15	11,790,307	0.13	0.07	0.21	5	11,292,846	0.04	0.01	0.10

*n*, number of operations; *CI*, confidence interval; *EVD*, external ventricular drain. Incidence refers to the incidence per 100,000 person-years calculated from the person-years column

**Fig. 2:**
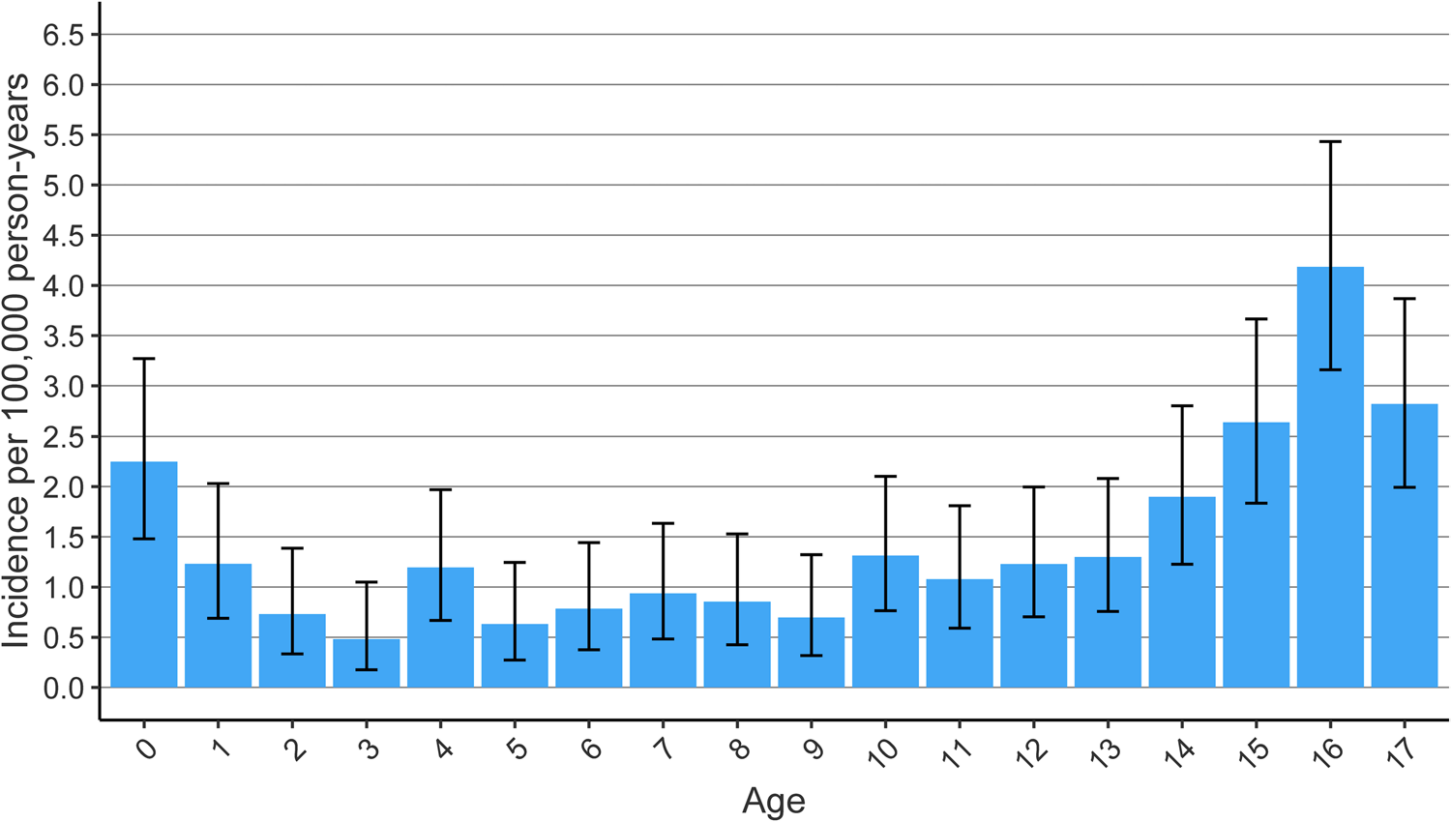
Cumulative incidence of neurosurgeries for pTBI over 20 years stratified by age per 100,000 person-years

The cumulative incidence of patients with pTBI undergoing neurosurgery was highest at the age of 16 (IRR4.18, CI3.16 to 5.43). The incidence was high at the age of 0 (IRR2.25, CI1.48 to 3.27) and declined considerably at the age of 1. The mean age was 10.6 years, and the median was 13 years. The incidence remained stable between ages 1 and 13 and increased after the age of 14 (see Fig. 2).

**Fig. 3:**
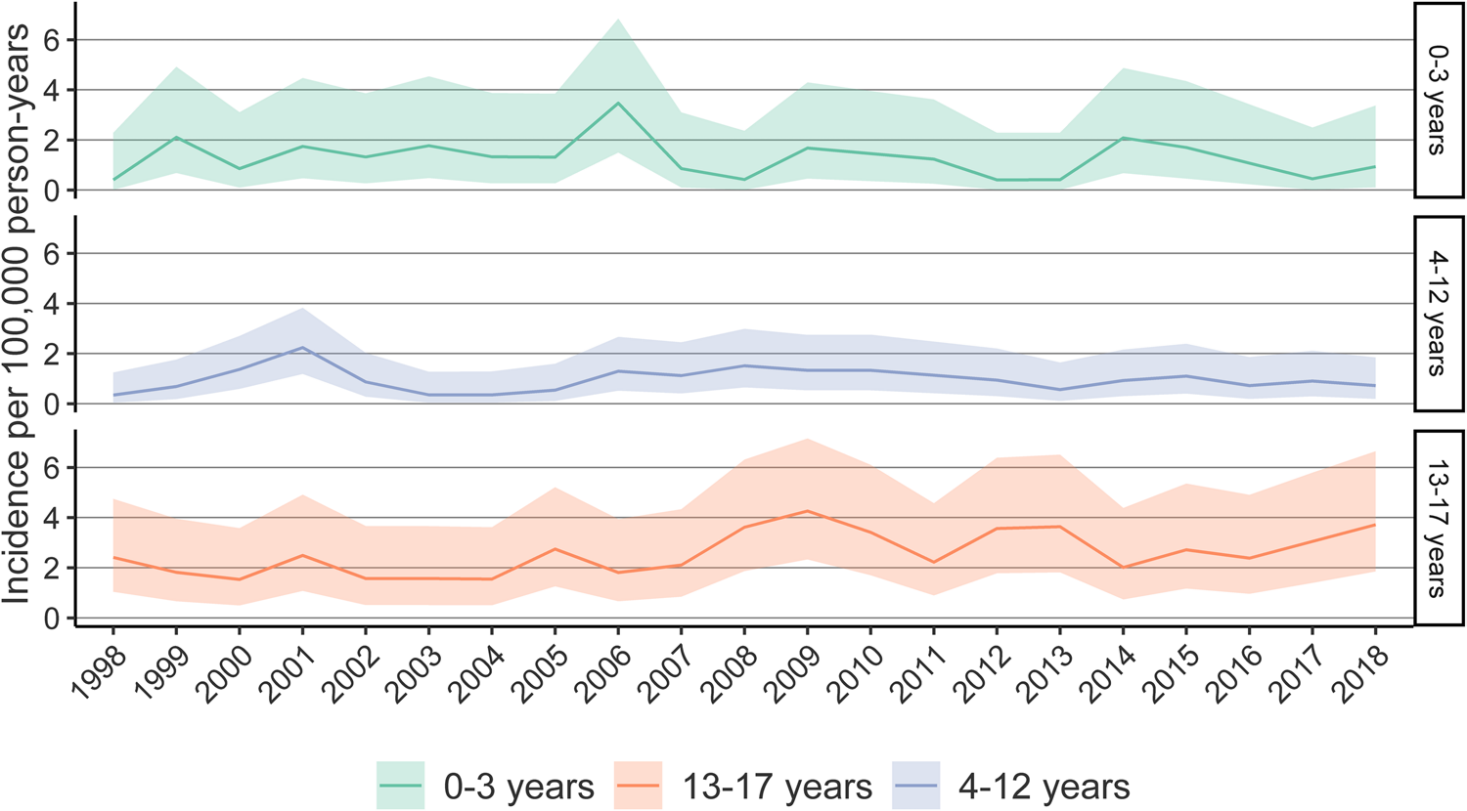
Incidence of neurosurgeries for pTBI by different age groups

In the 0- to 3-year age group, the cumulative incidence was 1.52 operations per 100,000 person-years (*n* = 56). In children 4 to 12 years of age, the cumulative incidence was 1.13 per 100,000 person-years (*n* = 112). The cumulative incidence in the 13 to 17 years of age group was 2.65 per 100,000 person-years (*n* = 173). No remarkable changes were observed in the incidence in any age-groups during the 21-year period (see Fig. 3).

**Fig. 4:**
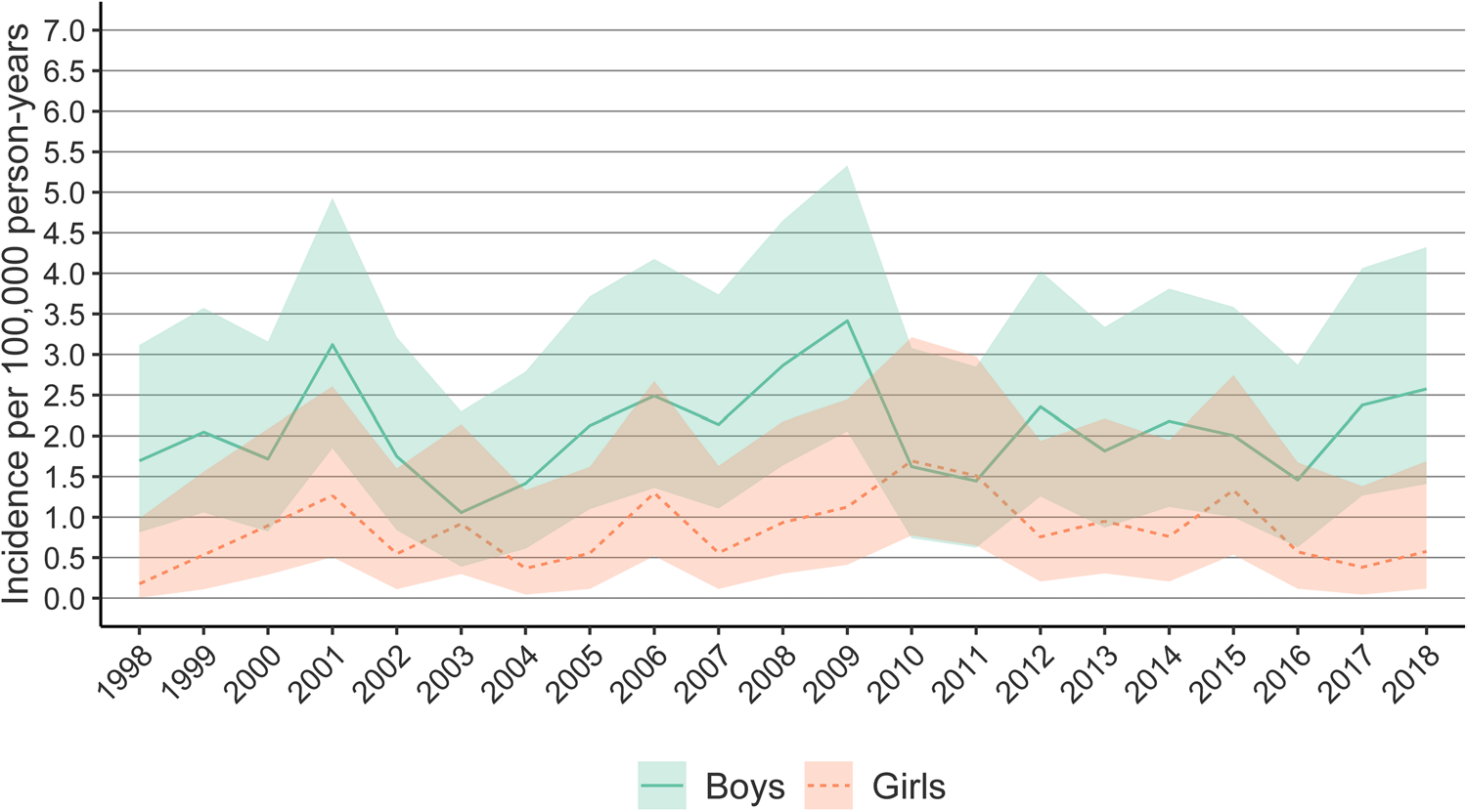
Incidence of neurosurgeries for pTBI by gender

Boys had 2.48 times higher cumulative incidence (IRR 2.08, CI 1.07 to 3.65, *n* = 246) than girls (IRR 0.84, CI 0.28 to 2.03, *n* = 95). Boys had higher yearly incidence every year except for 2010 and 2011 (see Fig. 4). The boys also had more operations for all different surgery types (see Table 1). The cumulative incidences for DCs and insertion of EVDs were notably higher for boys between the ages of 4 and 12 and for boys between the ages of 13 and 17 for insertions of EVDs and evacuations of epidural hemorrhages (see Table 1).

**Fig. 5:**
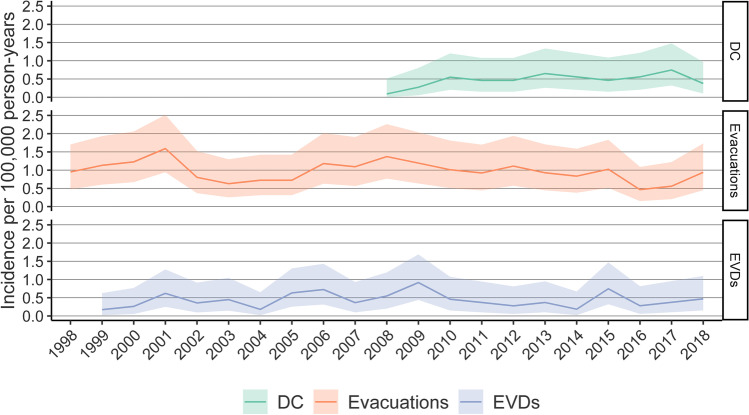
Incidence of DC (decompressive craniectomy), evacuations (intracranial hemorrhage evacuations), and EVDs (installation of external ventricular drains) during the years 1998 to 2018 in Finland

During the 20-year period, there were a total of 56 (14.8%) DCs, 96 (25.4%) insertions of EVDs, 168 (44.4%) evacuations of EDHs, 37 (9.8%) evacuations of acute SDHs, and 20 (5.3%) evacuations of intracerebral hemorrhage (see Table 1). For EVD insertions and intracranial hemorrhage evacuations, the incidence remained stable during the follow-up (see Fig. 5). No DCs were recorded before the year 2008 (see Fig. 5). For girls 0 to 3 years of age, no DCs were recorded at all (Table 1). The highest incidence of DCs was observed in 2017 (IRR 0.75, CI 0.32 to 1.47) (see Fig. 5). Boys had a higher incidence of EVD insertions, DCs, and evacuation of epidural hemorrhages than girls (see Table 1). The incidence of different operations increased with age (Table 1). The most frequent neurosurgical combination was insertion of EVD followed by DC (see Table 2).

**Table 2:** Number of different combinations of neurosurgery during 20 years of follow-up.

Combinations of neurosurgery	*n*
EVD + DC	12
Evacuation of aSDH + DC	4
Evacuation of EDH + DC	4
Evacuation of EDH + EVD	3
Evacuation of EDH + aSDH	2
Evacuation of intracerebral hemorrhage + EVD	2
EVD + DC + evacuation of EDH	2
Evacuation of aSDH + evacuation of intracerebral hemorrhage	1
DC + evacuation of intracerebral hemorrhage + evacuation of aSDH	1
Evacuation of aSDH + evacuation of EDH + DC + EVD	1

*n*, number of operations; *EVD*, external ventricular drain; *DC*, decompressive craniectomy; *aSDH*, acute subdural hemorrhage; *EDH*, epidural hemorrhage

## Discussion

We found that the incidence of neurosurgeries for pTBI has remained relatively stable from 1998 to 2018 in Finland. Boys had a higher incidence of operations, and most of the operations were performed on children less than 1 year of age or more than 12 years of age. International guidelines for the treatment of severe pTBI were first introduced in 2003 and have been evenly updated since. The overall trend of the incidence in operatively treated pTBI follows the guideline updates that have mostly concerned conservative treatment and monitoring while the role and criteria of operative treatment has stayed fairly the same. The Finnish national TBI management guidelines do not include children under the age of 16, so Finnish pediatric treatment follows mostly international guidelines [21, 23, 39].

In the Finnish pediatric population, the incidence of diagnosed pTBIs during a 21-year retrospective cohort study (1998–2018) was 332 per 100,000 person-years [25]. In our study, the cumulative incidence of acute neurosurgeries for these injuries was 1.47 per 100,000 person-years, which is 1.5% of all pTBIs in Finland. This is within the range of 0.7 to 10.5% of neurosurgeries for hospitalized pTBIs in industrial countries [2, 8, 9, 20, 31]. Most of these studies also included ICP-probe installations and fracture-elevations as operations [2, 8, 9, 20, 31]. Our data did not include ICP-probe installations or fracture elevations; therefore, our incidence might be modestly underestimated compared to the other studies. The incidence of pTBI-related neurosurgeries was fairly consistent over the years that follows the reported trend of moderate and severe pTBI incidences remaining stable [6, 13, 16, 35].

We observed peaks of incidence in ages 0 to 1 and 14 to 17. In worldwide analyses, this bimodal age distribution of pTBIs is also usually seen for the ages 0 to 2 and 15 to 18 [11]. The fact that the incidence was higher in toddlers than preschool/elementary school-age children might be explained by toddlers falling more and adolescents tendency of doing higher-risk activities (e.g., motorcycling and contact sports). The cumulative incidence for boys was higher than for girls in the 4 to 12 and 13 to 17 years of age groups. This is consistent with global research on pTBI where the incidence between genders is similar through ages 0 to 4 and increases for boys after pre-school age [2, 11, 12, 24]. According to a prior Finnish study, boys were 1.5 times more likely to sustain a pTBI. In the present study, boys were 2.5 times more likely to undergo a pTBI-related operation [35]. Boys are more likely to have more severe pTBIs that are more likely to require surgery [34]. The higher incidence rates for boys could be explained in part by the more physical nature of their childhood play, greater attendance rates in sports, and more risk-taking behavior.

The two most frequent operation types in Finland were evacuation of intracranial hemorrhage (60%) and the insertion of EVDs (25%). The proportion of intracranial hemorrhage evacuations is higher than the global range of 32 to 48%, but EVD insertions are within the global range of 18 to 47% [2, 9, 28]. The global range of EVDs also includes ICP probe insertions; therefore, our rate is likely underestimated. Our higher proportional rate of intracranial hemorrhage evacuations might be due to the fact that we did not study fracture-related operations, and these procedures correspond to 13 to 23% of all operations in global data [2, 9, 28]. The limited use of EVDs compared to the intracranial hemorrhage evacuations could also be explained by the lack of sufficient quality studies and the large variation across and within hospitals regarding cerebrospinal fluid diversion strategies [4]. Simultaneous fracture repairs are often performed as part of hemorrhage evacuations because fractures and intracranial lesions are often located in the same anatomical region. Therefore, operation codes can be missing for fracture repairs that are done on the side of acute intracranial neurosurgery. Our study sample contains likely some cases that had cranial fracture surgery as part of the evacuation of an intracranial hemorrhage that have not been recorded.

There were no documented decompressive craniectomies for treating severe pTBI with uncontrollable high ICP before the year 2008, and the incidence rates of DC have slightly increased thereafter. The guideline update released in 2008, in addition to few studies released in 2007 to 2008, suggested that DC could have major benefit on early treatment of uncontrollably high ICP in patients who are carefully selected [5, 14, 19]. This might explain why the incidence of DC has been increasing since 2008 in Finland. The most recent studies have supported the role of DC in a selected group of patients, but there still are no clearly defined guidelines on this topic [15, 17, 22, 36].

A major strength of our study was the access to the Finnish Care Register for Healthcare that includes all visits and operations nationwide and has information regarding all healthcare levels from primary and secondary to the tertiary level. This register has shown great coverage and accuracy for containing ICD-10 codes and operation codes [18, 27]. This large and reliable database supports the generalizability of our findings to the Finnish pediatric population. Finland has a very established practice of using ICD-10 codes and operation codes in every visit, so it is unlikely that our data is missing any visits or operations. A weakness of our study is the lack of information on the injury mechanisms and the severity of the TBI, and while the operation codes are descriptive and clear, some operations that have multiple phases could have missing operation codes. In addition, we excluded skull fracture repairs that may affect the generalizability of our results on global research.

## Conclusion

The incidence of neurosurgeries for pTBIs has been relatively stable during the last 2 decades in Finland. Boys were more likely to undergo an operation after pTBI than girls. The incidence of neurosurgery for pTBI is highest among babies and teenagers. The most frequent lesion that needed neurosurgery was epidural hemorrhage. The only operation type that showed an increase in the incidence during the study period was decompressive craniectomy.

## Supplementary Information

Below is the link to the electronic supplementary material.

Supplementary Table (DOCX 15.4 kb)

## Abbreviations

pTBI: Pediatric traumatic brain injury
USA: United States of America
TBI: Traumatic brain injury
EDH: Epidural hemorrhage
SDH: Subdural hemorrhage
SAH: Subarachnoid hemorrhage
ICP: Intracranial pressure
EVD: External ventricular drain
DC: Decompressive craniotomy
ICD-10: International Classification of Diseases 10th Edition
CI: Confidence interval
IRR: Incidence rate ratio
