# Supplementary material for: Incidence of acute neurosurgery for traumatic brain injury in children—a nationwide analysis from 1998 to 2018
Source: Acta Neurochir (Wien). 2023 May 15;165(7):2001–9. doi: 10.1007/s00701-023-05628-0 (PMC10319660; doi:10.1007/s00701-023-05628-0)
Supplement: Supplementary file 1 — (DOCX 15.4 kb) [file 701_2023_5628_MOESM1_ESM.docx]

**Appendix 1. Tables containing ICD-10 Diagnose codes and operation codes used in the study.**

Incidence of acute neurosurgery for traumatic brain injury in children – a nationwide analysis from 1998 to 2018

**Authors:**

Julius Möttönen BM^1,2^; Ville T Ponkilainen MD, PhD^4^; Grant L Iverson PhD^5,6,7,8^; Teemu Luoto MD, PhD^2,9^; Ville M Mattila MD, PhD^2,10^; Ilari Kuitunen MD, PhD^1,3^

**Affiliations:**

1 Institute of Clinical Medicine, University of Eastern Finland, Kuopio, Finland

2 Tampere University, Faculty of Medicine and Life Sciences, Tampere, Finland

3 Department of Pediatrics, Mikkeli Central Hospital, Mikkeli, Finland

4 Department of Surgery, Central Finland Hospital Nova, Jyväskylä, Finland

5 Department of Physical Medicine and Rehabilitation, Harvard Medical School, Boston, MA, USA

6 Department of Physical Medicine and Rehabilitation, Spaulding Rehabilitation Hospital, Charlestown, MA, United States

7 Department of Physical Medicine and Rehabilitation, Schoen Adams Research Institute at Spaulding Rehabilitation, Charlestown, MA, United States

8 Sports Concussion Program, MassGeneral Hospital for Children, Boston, MA, United States

9 Department of Neurosurgery, Tampere University Hospital, Tampere, Finland

10 Tampere University Hospital, Department of Orthopedics and Traumatology, Tampere, Finland

**Corresponding author:**

Julius Möttönen, e-mail: julius.mottonen@tuni.fi, address: Tampere University, Kalevantie 4 33100 Tampere, ORCID: 0000-0001-7366-5140.

Table 1. Diagnose codes (ICD-10 diagnostic code system).

| Diagnose code | Definition |
| --- | --- |
| S06.0 | Concussion |
| S06.1 | Traumatic cerebral edema |
| S06.2 | Diffuse traumatic brain injury |
| S06.3 | Focal traumatic brain injury |
| S06.4 | Epidural hemorrhage |
| S06.5 | Traumatic subdural hemorrhage |
| S06.6 | Traumatic subarachnoid hemorrhage |
| S06.7 | Intracranial injury and prolonged concussion |
| S06.8 | Other intracranial injury |
| S06.9 | Unspecified intracranial injury |

Table 2. Operation codes (NCSP Finnish version).

| Operation code | Definition |
| --- | --- |
| AAD00 | Evacuation of epidural hematoma |
| AAD05 | Evacuation of acute subdural hematoma |
| AAD15 | Evacuation of traumatic intracerebral hematoma |
| AAF00 | Ventriculostomy |
| AAK80 | Partial excision of skull cap for relief of acute cerebral edema |
